# Effects of Dietary Crude Protein Level of Concentrate Mix on Growth Performance, Rumen Characteristics, Blood Metabolites, and Methane Emissions in Fattening Hanwoo Steers

**DOI:** 10.3390/ani14030469

**Published:** 2024-01-31

**Authors:** Joonpyo Oh, Hyunjin Cho, Sinyong Jeong, Kyewon Kang, Mingyung Lee, Seoyoung Jeon, Hamin Kang, Seongwon Seo

**Affiliations:** 1 Cargill Animal Nutrition Korea, Seongnam 13630, Republic of Korea; davidoh@cargill.com; 2 Division of Animal and Dairy Sciences, Chungnam National University, Daejeon 34134, Republic of Korea; chohyunjin0927@gmail.com (H.C.); syjeong89@gmail.com (S.J.); kangkyewon26@gmail.com (K.K.); mingyung1203@gmail.com (M.L.); seoyoung203@gmail.com (S.J.); gkals0339@gmail.com (H.K.)

**Keywords:** crude protein, methane, laser methane detector, Hanwoo

## Abstract

**Simple Summary:**

This study investigated the effect of varying the levels of dietary crude protein (CP) in concentrate mixes on growth performance, rumen characteristics, digestibility, blood metabolites, and methane emissions in fattening Hanwoo steers. The experiment was conducted for 12 weeks by feeding four concentrate mixes with different levels of dietary CP (15, 18, 19, and 21% of CP dry matter basis). There was a linear increasing trend in the average daily gain (ADG) with increasing dietary CP (*p* = 0.066). The increase in CP levels also influenced the characteristics of the rumen fluid, leading to increased concentrations of rumen ammonia and an increase in the proportion of butyrate and valerate (*p* < 0.05) while decreasing the propionate proportion (*p* = 0.004). The blood urea increased (*p* < 0.001) and the blood non-esterified fatty acids and cholesterol decreased (*p* ≤ 0.003) as the dietary CP increased. The methane concentration from eructation per intake (ppm/kg), forage neutral detergent fiber (NDF) intake, total NDF intake, and ADG exhibited linear decreases (*p* ≤ 0.014) across diets. In summary, increasing the dietary CP to 21% in concentrates tended to increase the ADG, reduce the propionate, and increase the butyrate. The methane from eructation showed a tendency to linearly decrease with higher CP.

**Abstract:**

This study aimed to investigate the effect of varying levels of dietary crude protein (CP) on growth performance, rumen characteristics, blood metabolites, and methane emissions in fattening Hanwoo steers. Twenty-four steers, weighing 504 ± 33.0 kg (16 months old), were assigned to four dietary treatments with different CP concentrations (15, 18, 19, and 21% of CP on a dry matter (DM) basis). A linear increasing trend in the average daily gain (ADG) was observed (*p* = 0.066). With increased dietary CP levels, the rumen ammonia concentration significantly increased (*p* < 0.001), while the propionate proportion linearly decreased (*p* = 0.004) and the proportions of butyrate and valerate linearly increased (*p* ≤ 0.003). The blood urea exhibited a linear increase (*p* < 0.001), whereas the blood non-esterified fatty acids and cholesterol showed a linear decrease (*p* ≤ 0.003) with increasing dietary CP. The methane concentration from eructation per intake (ppm/kg), forage neutral detergent fiber (NDF) intake, total NDF intake, and ADG exhibited linear decreases (*p* ≤ 0.014) across the treatments. In conclusion, increasing the dietary CP up to 21% in concentrates demonstrated a tendency to linearly increase the ADG and significantly decrease the propionate while increasing the butyrate. The methane concentration from eructation exhibited a tendency to linearly decrease with increasing dietary CP.

## 1. Introduction

Feeding appropriate crude protein (CP) levels is very important in beef cattle production, considering its effects on productivity. A deficiency in dietary protein in beef cattle may result in negative effects on weight gain, feed intake, and carcass results [1].

Meanwhile, an excessive CP supply increases the energy expenditure of removing excessive nitrogen as urea and also causes a detrimental impact on the environment by increasing nitrogen excretion [2,3].

Studies have been conducted to investigate the effects of various CP levels on the production and body metabolism, including ruminal fermentation and blood metabolites, in beef cattle. Gleghorn et al. [4] reported that increasing dietary CP from 11.5 to 14.5% dry matter (DM) quadratically increased the average daily gain (ADG) and hot carcass weight in finishing beef steers. In another study with young Holestein bulls, increasing CP from 10.2 to 14.2% DM linearly increased the ADG and feed efficiency [5]. Increasing CP modified ruminal fermentation by increasing ammonia (NH_3_-N), acetate, and propionate concentrations and increased the blood urea nitrogen concentration in the same study [5]. Additionally, dietary CP levels were reported to affect enteric methane (CH_4_) production in ruminants, although the results were inconsistent between studies [6,7].

The effects of dietary CP levels were also investigated in Hanwoo cattle, which is a native Korean breed. Jeong et al. [8] found that higher CP increased the ADG in late-fattening (23 to 30 months) Hanwoo steers fed an iso-energetic diet. However, other studies reported no effect of increasing CP on weight gain in growing and finishing steers [9,10]. The carcass weight of Hanwoo beef cattle has been continuously improved by the genetic selection program run by the Korean government [11]. According to a study reported by the Korea Institute for Animal Products Quality Evaluation, the national average carcass weight in Hanwoo steers has improved by 11.0% over the past ten years [12]. It is certain that studies are needed to investigate the effects of dietary CP levels on productivity and body metabolism in Hanwoo steers because of genetic improvement. However, there are limited studies about the effects of dietary CP levels in Hanwoo steers in the fattening stage. Therefore, the objective of this study was to investigate the effects of different levels of dietary CP on the growth performance, rumen characteristics, blood metabolites, and CH_4_ emissions of Hanwoo fattening steers. We hypothesized that an increase in the CP level of the concentrate mix would enhance productivity and have no impact on CH_4_ emissions.

## 2. Materials and Methods

This study was conducted in the Animal Science Research Center at Chungnam National University in Korea. The use of animals and the protocols for this experiment were reviewed and pre-approved by the Chungnam National University Animal Research Ethics Committee (202203A-CNU-057).

### 2.1. Animals, Housing, and Diets

Twenty-four 16-month-old Hanwoo fattening (15 to 22 months) steers (504 ± 33.0 kg), which were blocked by initial body weight (BW) and estimated breeding values for carcass BW, were randomly assigned to one of four dietary treatments based on a completely randomized block design [13]. Within each block, two steers with similar BW were paired and housed together in a pen (5 m × 5 m). Each pen was equipped with a forage feed bunk (Dawoon, Co., Incheon, Republic of Korea) that utilized a radio-frequency identification (RFID) tag to automatically measure the individual feed intake of the steers. The forage feed bunk incorporated two load cells for assessing the amount of feed remaining, sensors to identify the presence of the steers, and a panel designed to detect the RFID neck tags of each steer. The system operated so that the panel would rise if a steer’s RFID neck tag was not detected and lower to allow access when the tag was recognized. This mechanism ensured that only one steer could access the feed bunk at a time. Additionally, the intake of concentrate mix for each steer was monitored manually. This involved measuring the quantity of concentrate mix fed to the steers and the residue left after feeding. The experiment was conducted for three months. Prior to the commencement of the experiment, the steers underwent a seven-day adaptation period to acclimate to the experimental environment and diets. Following the adaptation period, the steers were fed the experimental diets for three months.

Two types of concentrate mix were prepared for the study: a low-CP concentrate mix containing 15% CP on a DM basis and a high-CP concentrate mix containing 21% CP on a DM basis. These were combined in varying proportions to create four dietary treatments with incremental CP levels: (1) low CP (LCP; 100% low-CP concentrate mix; 15% CP on a DM basis), (2) medium-low CP (MLCP; 50% low-CP concentrate mix and 50% high-CP concentrate mix; 18% CP on a DM basis), (3) medium-high CP (MHCP; 25% low-CP concentrate mix and 75% high-CP concentrate mix; 19% CP on a DM basis), and (4) high CP (HCP; 100% high-CP concentrate mix; 21% CP on a DM basis). The diets aimed for a target average daily gain (ADG) of 0.9 kg/day, in accordance with the Korean feeding standards for Hanwoo growing steers [14]. Feedings were scheduled at 0800 and 1800 h. Tall fescue, which was provided as forage, and drinking water were available ad libitum throughout the experiment. Detailed formulations and chemical compositions of the experimental diets are presented in Table 1 and Table 2.

### 2.2. Measurement and Sample Collection

The daily intake of concentrate mix for each steer was manually recorded, while forage intake was automatically recorded by a forage feed bunk. Every four weeks, feed intakes were evaluated, and values that were more than or less than three standard deviations from the mean were excluded as outliers. Intake affected by disruptions, like bedding changes, body weight measurements, and sampling, were also excluded. The steers’ body weight and feed samples for chemical analysis were collected every four weeks.

Rumen fluid was collected three times (−1, +3, and +6 h after morning feeding) on three consecutive days during weeks 5 and 11 using an oral stomach tube as described in Lee et al. [15]. Briefly, 400 mL of rumen fluid was collected in a glass flask after the initially collected samples were discarded (approximately 300 mL of rumen fluid). The pH of the rumen samples was immediately measured, and 10 mL of each was subsampled and stored at −20 °C until analysis for NH_3_-N and volatile fatty acid (VFA).

About 10 mL of blood was collected once from the jugular vein of all steers before morning feeding during week 12 using serum separator tubes (BD Vacutainer; BD and Co., Franklin Lakes, NJ, USA). Blood serum was obtained by centrifugation at 1300× *g* for 15 min at 4 °C and frozen at –80 °C until analysis for blood metabolites.

Methane emissions of all steers were measured four times (−2, −1, +1, and +2 h after morning feeding) for five consecutive days using a laser methane detector (LMD; LMD; Laser Methane Mini, Tokyo Gas Engineering Solutions Co., Ltd., Tokyo, Japan) during week 10 as described by Kang et al. [16] and duplicated for an additional five consecutive days during week 11. Briefly, with the LMD installed stably on a tripod, the visible laser was aimed at the steer’s nose from a distance of 1 m, and CH_4_ emissions were measured every 0.5 s for 6 min.

### 2.3. Sample Analyses

The feed samples were dried at 60 °C for 96 h in an air-forced dry oven and ground through a cyclone mill (Foss, Hillerød, Denmark) fitted with a 1 mm screen. The nutrient composition of the feed samples was analyzed at Cumberland Valley Analytical Services Inc (Hagerstown, MD, USA). Details of the methods used to analyze the nutrient contents in the feeds were the same as described in Jeon et al. [17].

The comprehensive details of NH_3_-N, VFA, and blood serum analyses are described in Cho et al. [18]. Briefly, to analyze the NH_3_-N concentration in rumen fluid, a process involving centrifugation and reaction with phenol color and alkali-hypochlorite reagents was used, followed by incubation in a water bath and spectrophotometric measurement at 630 nm using a spectrophotometer (UV-1800, Shimadzu, Kyoto, Japan). For the VFA concentration determination, the rumen fluid supernatant was mixed with metaphosphoric acid, cooled, centrifuged, and then analyzed using a Hewlett-Packard HP 6890 gas chromatograph (Hewlett Packard Co., Palo Alto, CA, USA) with specific temperature settings and a flame ionization detector (Nukol Fused silica capillary column 30 m × 0.25 mm × 0.2 μm, Supelco Inc., Bellefonte, PA, USA). Furthermore, serum analysis for various blood metabolites was conducted using commercial kits and a clinical auto-analyzer (Toshiba Accute Biochemical Analyzer-TBA-40FR, Toshiba Medical Instruments, Tokyo, Japan).

Methane emission data were divided into respiration and eructation by identifying CH_4_ concentration peaks using an automated peak detection package in R software [19] version 4.0.2. These peaks were then analyzed using a double normal distribution approach with the mixdist package in R. This model’s mean value represented the average daily CH_4_ concentration, which was calculated as the mean of CH_4_ concentrations at four different times of the day. These values were considered indicative of the CH_4_ levels in the exhaled gas from the respective pathways during these specific periods [16].

### 2.4. Statistical Analysis

All data were analyzed using the PROC MIXED procedure of SAS (SAS Institute Inc., Cary, NC, USA), as recommended by Seo et al. [13]. The blocks (i.e., initial BW and breeding value for carcass weight) were treated as random effects. Individual means were also compared by Tukey’s test. Additionally, linear and quadratic effects were assessed by orthogonal contrasts. To evaluate the impact of treatments on rumen parameters, the study employed a repeated measures analysis. This approach was chosen to acknowledge the correlations between multiple observations taken from each animal. In this analysis, no structure was assumed for the variance–covariance matrix. Statistical significance was established at a *p*-value of less than 0.05, while observations with a *p*-value between 0.05 and 0.1 were considered indicative of a trend.

## 3. Results and Discussion

The animal performance data are shown in Table 3. The initial and final BW, dry matter intake (DMI) of the concentrate and forage, forage/concentrate ratio, and feed conversion ratio were similar between the treatments. However, we found a trend of linear increase (*p* = 0.066) in the ADG. As expected, the CP intake was linearly increased (*p* < 0.001) by the treatments, while the net energy for growth intake was similar. There was no difference in the pH between the treatments (Table 4), but the ruminal NH_3_-N concentration significantly increased (*p* < 0.001) with increasing dietary CP levels. The total VFA concentration and molar proportion of acetate were not different between the treatments, but the propionate proportion was linearly decreased (*p* = 0.004) with increased CP. This resulted in a linear decrease (*p* = 0.030) in the acetate/propionate ratio. The molar proportions of butyrate and valerate linearly increased (*p* ≤ 0.003) with the treatments. The blood concentrations of total protein, glucose, albumin, creatinine, triglyceride, glutamic oxaloacetic transaminase, glutamic pyruvic transaminase, calcium, and phosphorus were not affected by the treatments (Table 5). The blood urea concentrations linearly increased (*p* ≤ 0.003), and the non-esterified fatty acid (NEFA) and cholesterol concentrations linearly decreased (*p* ≤ 0.003) in the blood with increasing CP levels. We found a trend of linear decrease (*p* = 0.063) in the CH_4_ concentration of the exhaled gas from eructation (Table 6). The CH_4_ concentration during eructation per DMI, forage neutral detergent fiber (NDF) intake, NDF intake, and ADG were linearly decreased (*p* ≤ 0.014) by the treatments. There was a quadratic increase (*p* = 0.049) in the CH_4_ concentration of the exhaled gas from respiration. The treatments tended to linearly decrease (*p* ≤ 0.086) the exhaled CH_4_ emissions per forage NDF intake and NDF intake.

It should be noted that terms referring to growth stages are different between studies because the production systems for beef cattle differ depending on feed resource availability, consumers’ needs in the market (e.g., high marbled beef), climate, geographical reason, characteristics of breed (e.g., body frame), and genetics [20]. For example, in Korea, native Hanwoo steers are typically slaughtered at around 30 months of age to produce high-marbled beef. In this context, the growth stage from 7 to 14 months of age is commonly referred to as the ‘growing phase’, 15 to 22 mo. as the ‘fattening phase’, and 23 to 30 mo. as the ‘finishing phase’. In the present study, steers with an average BW of 504 kg in the fattening phase are comparable with those in the finishing phase of feedlot systems in other countries, including North and South America and Europe.

Studies have shown that increasing the CP level did not affect the feed intake in beef cattle. Boonsaen et al. [21] reported no difference in the DMI when feedlot steers were fed total mixed ratio (TMR) with 12 or 14% CP in a 120 d feeding trial, which is consistent with the current study. Gleghorn et al. [4] also found that the DMI was not affected by a CP increase from 11.5 to 14.5% in concentrates in cross-bred feedlot steers. There are limited studies on Hanwoo steers. Recently, Jeon et al. [22] reported that feeding with 22.2% CP concentrate did not affect the DMI compared with 19.5% in growing Hanwoo steers. The intake response to the CP level may differ between growth stages. According to Bailey et al. [23], increasing the concentrate CP from 11 to 14.0% did not affect the DMI in the growing phase, but quadratically increased in the finishing phase. The authors speculated that supplying ruminally degradable protein (RDP) in an 11% concentrate CP diet in the finishing phase limited and reduced microbial efficiency, leading to intake reduction [23]. Reduced feed intake has been reported in dairy cattle fed MP-deficient diets [24]. However, all the treatments were formulated to meet the protein requirement in the current study, which resulted in no difference in DMI.

Data about the effects of dietary CP level on weight gain in steers varied between studies. A higher ADG was reported in finishing bulls fed 15.0% CP TMR compared with 13.5% in a 162 d feeding trial [25]. Because the feed conversion ratio (FCR) was similar between treatments, the authors concluded that the positive result in the ADG for the higher CP group was attributed to higher DMI compared with bulls fed lower CP TMR. Similar results were found in a study with finishing steers by Archibeque et al. [26], where the ADG and gain-to-feed ratio were improved by both the medium (11.8% CP) and high (14.9% CP) groups compared with the low (9.1% CP) group. It was not discussed how the medium and high groups increased the ADG and gain-to-feed ratio and whether the 9.1% CP diet met the MP requirement of the low group in that study. However, there was a trend of increase in the DMI for the medium and high groups, which possibly led to positive effects on the production data. These studies are partially in line with the results of the current study since we observed a trend of increase in the ADG, but not for the DMI or FCR. Similar results to the current study were found in a study by Bailey et al. [23], who reported that increasing CP concentration (11, 12.5, and 14.0%) linearly increased the ADG without effects on the DMI in growing cattle, including steers and heifers. In the same study, however, responses to the CP levels were different in finishing cattle, that is, the ADG was quadratically increased, along with a quadratic increase in the DMI [23]. Gleghorn et al. [4] also found a quadratic increase in the ADG but no effect on the DMI, with increasing CP levels (11.5, 13.0, and 14.5%) in finishing steers, which is also consistent with the results of the ADG and DMI in the current study. As previously mentioned, the diets in the current study were formulated to meet the nutritional requirements according to Korean feeding standards for Hanwoo steers targeting 900 g of ADG [14]. However, the ADG in all the treatment groups was less than 900 g in the current study.

The ruminal pHs in the current study were comparable with the ones in a similar study with Hanwoo steers [27]. A linear increase in the ruminal NH_3_-N concentration in the current study is in agreement with other studies in beef cattle [5,28,29]. This indicates that increasing the CP in concentrates up to 19% as-is in Hanwoo steers could result in excessive ruminal NH_3_-N and increase the conversion into urea in the liver and excretion of urea in urine. The effects of increasing the CP on ruminal VFA concentrations are not consistent between studies. Some studies in beef and dairy cattle reported no effects of increasing CP on ruminal VFA concentrations [28,30]. Chanthakhoun et al. [29] found a linear increase in the propionate concentration with a concentrate CP range of 9.2 to 21.9% in buffaloes, which is inconsistent with the current study. Changes in the VFA concentrations were probably due to content differences in carbohydrates in diets. The NFC content numerically decreased with increasing CP in the current study, which likely caused the linear decrease in the propionate concentration in the rumen. The linear increase in butyrate in the present study was not in agreement with other studies. Oh et al. [27] and Chen et al. [28] observed no effect of increasing CP on ruminal butyrate concentration. Brandao and Faciola [31] also reported no relationship between dietary CP and ruminal butyrate in a meta-analysis study using a dual-flow continuous culture system. It is likely that the nutrient composition of diets in the current study might increase the butyrate concentration in the rumen. Butyrate-producing bacteria, such as *Butyrivibrio* Spp., could be increased by the fiber amount, and the NDF amount was numerically higher in higher-CP diets in the current study [32]. Valerate is one of the branched chain fatty acids, which are by-products of the deamination of amino acids in the rumen [33]. It is possibly thought that valerate was produced more in higher-CP diets, which is consistent with the NH_3_-N results in the current study.

It has been reported that increasing CP levels enhanced the blood urea nitrogen concentration in studies with beef cattle [5,29]. This is in agreement with the current study, and it could be speculated that excessive ruminal NH_3_-N resulted in increased urea concentration in blood. The concentrations of blood NEFA in the current study were comparable with those in a study with Hanwoo steers [34]. It is unclear how increasing CP decreased the blood NEFA in the current study. Bharanidharan et al. [35] found no difference in the blood NEFA between two different dietary CP levels in Hanwoo steers. Decreases in the blood cholesterol concentration with increasing dietary CP were also found in other studies with growing calves and beef steers [9,36]. Park [36] demonstrated that a higher protein diet increased lecithin cholesterol acyltransferase activity compared with a lower protein diet, which relates to the cholesterol lipid distribution in the liver.

The effects of increasing (or decreasing) the CP concentration in the diet on enteric CH_4_ production in ruminants have been inconsistent between studies. Hynes et al. [7] found no effect of increasing CP (14.1 to 18.1% DM) in diets on CH_4_ emissions in dairy cattle. Kidane et al. [37] also reported that increasing dietary CP (13.0 to 17.5% DM) did not affect CH_4_ emissions in dairy cattle. However, Arndt et al. [6] observed quadratic increases in the CH_4_ emissions (g/d) and emission yield (g/kg of DMI) with increasing dietary CP (16.6 to 18.0% DM) in different ratios of alfalfa silage to corn silage. In contrast, in a meta-analysis study, dietary CP concentration had a negative relationship with the CH_4_ emission yield (g/kg of DMI) in dairy cows [38]. This is in agreement with the current study’s findings showing that increasing the CP content linearly decreased the CH_4_ concentration and CH_4_ concentration/kg DMI. One should carefully interpret these results because ruminal VFA concentrations (decrease in the propionate and increase in the butyrate) in the current study were not supportive of the CH_4_ data. It is unclear how the ruminal CH_4_ concentration was negatively affected by the CP content in the present study. Possibly, higher inclusion of distiller’s grains with solubles (DDGS) might decrease the CH_4_ concentration, similar to other studies in dairy and beef cattle [39,40,41]. It is known that DDGS contains a relatively high PUFA concentration [42], and unsaturated fatty acids have a negative effect on CH_4_ formation by eliminating hydrogens in the rumen. However, the fatty acid composition in diets was not analyzed in the current study.

## 4. Conclusions

In conclusion, increasing the dietary CP up to 21% as-is in concentrates had a tendency of linear increase in the ADG with no effect on the DMI in Hanwoo fattening steers. In addition, the treatments modified ruminal fermentation by decreasing the propionate concentration and increasing the butyrate concentration. The enteric CH_4_ concentration from eructation also tended to decrease when increasing the CP in the current study.

## Figures and Tables

**Table 1:** Diet formulation of the experimental concentrate mix.

Items ^1^	Treatment ^2^			
	LCP	MLCP	MHCP	HCP
Ingredients (g/kg DM)				
Corn, flaked	192	192	192	192
Corn, ground	57	31	18	5
Wheat, ground	140	157	165	173
Hydrogenated fat ^3^	23	12	6	0
Palm oil	11	10	9	9
Corn gluten feed	159	159	159	159
Soybean hull	98	49	25	0
Wheat bran	63	32	16	0
Molasses	44	31	24	18
DDGS	50	172	233	294
Palm kernel meal	103	101	100	100
CMS	13	13	13	13
Urea	0	3	5	6
Ammonium chloride	2	2	2	2
Limestone	32	25	22	19
Salt	2	2	2	2
Vitamin and mineral mix *	8	8	8	8

^1^ DDGS, distiller’s grains with solubles; CMS, condensed molasses solubles. ^2^ LCP, low crude protein (15%); MLCP, middle-low crude protein (18%); MHCP, middle-high crude protein (19%); HCP, high crude protein (21%). ^3^ It is a hydrogenated free fatty acid mainly composed of C16 (56.0%) and C18 (42.0%) and it was used to complement the energy in the feed. * 33,330,000 IU/kg vitamin A, 40,000,000 IU/kg vitamin D, 20.86 IU/kg vitamin E, 20 mg/kg Cu, 90 mg/kg Mn, 100 mg/kg Zn, 250 mg/kg Fe, 0.4 mg/kg I, and 0.4 mg/kg Se.

**Table 2:** Chemical composition (g/kg DM or as stated) of the experimental diets.

Items ^1^	Treatment ^2^				Tall Fescue
	LCP	MLCP	MHCP	HCP	
DM, g/kg as fed	886	886	886	886	888
OM	911	916	918	920	941
CP	147	178	193	208	69
SOLP	58	67	72	77	30
NDICP	26	27	27	27	13
ADICP	12	13	14	15	9
aNDF	271	286	293	301	640
ADF	134	131	129	128	404
ADL	38	37	36	36	62
Ether extract	69	62	59	56	13
Ash	89	84	82	80	59
Ca	15	13	12	10	3
P	6	6	7	7	1
K	10	11	11	12	18
Na	2	2	3	3	1
Cl	5	5	5	5	5
S	4	4	5	5	1
Mg	3	4	4	4	1
TDNs	747	740	736	733	557
NEm, MJ/kg DM	8.1	8.0	7.9	7.8	5.0
NEg, MJ/kg DM	5.4	5.3	5.2	5.2	2.7
Total carbohydrates	695	676	666	656	859
NFC	450	417	400	383	232
Carbohydrate fraction, g/kg carbohydrate					
CA	84	88	91	93	127
CB1	554	506	481	457	2
CB2	9	21	27	34	141
CB3	221	253	269	285	556
CC	132	131	131	131	174
Protein fraction, g/kg CP					
PA+B1	391	381	376	371	435
PB2	431	463	480	496	374
PB3	98	80	70	61	64
PC	79	76	74	72	128

^1^ DM: dry matter; OM: organic matter; CP: crude protein; SOLP: soluble CP; NDICP: neutral detergent insoluble CP; ADICP: acid detergent insoluble CP; aNDF: neutral detergent fiber analyzed using a heat-stable amylase and expressed inclusive of residual ash; ADF: acid detergent fiber; ADL: acid detergent lignin; TDNs: total digestible nutrients; NEm: net energy for maintenance; NEg: net energy for growth; NFC: non-fiber carbohydrate; CA: carbohydrate A fraction; ethanol soluble carbohydrate, CB1: carbohydrate B1 fraction; starch, CB2: carbohydrate B2 fraction; soluble fiber, CB3: carbohydrate B3 fraction; available insoluble fiber, CC: carbohydrate C fraction; unavailable carbohydrate, PA+B1: protein A and B1 fractions; soluble CP, PB2: protein B2 fraction; intermediate degradable CP, PB3: protein B3 fraction; slowly degradable fiber-bound CP, PC: protein C fraction; unavailable CP. ^2^ LCP, low crude protein (15%); MLCP, middle-low crude protein (18%); MHCP, middle-high crude protein (19%); HCP, high crude protein (21%).

**Table 3:** Effects of dietary crude protein level in concentrate mix on growth performance.

Items ^1^	Treatment ^2^				SEM	*p*-Value		
	LCP	MLCP	MHCP	HCP		Mean	Linear	Quadratic
Initial BW, kg	501	500	504	507	5.7	0.819	0.483	0.540
Final BW, kg	564	569	570	579	6.7	0.426	0.140	0.564
ADG, g/day	617	675	647	708	31.8	0.182	0.066	0.897
DMI, kg/day								
Concentrate	6.14	6.55	6.41	6.35	0.170	0.364	0.310	0.165
Forage	2.43	2.34	2.61	2.56	0.167	0.628	0.406	0.664
Total	8.57	8.89	9.02	8.90	0.214	0.409	0.154	0.433
Forage/concentrate	0.40	0.36	0.41	0.40	0.029	0.603	0.701	0.453
CP intake, kg/day	1.07 ^c^	1.34 ^b^	1.42 ^ab^	1.50 ^a^	0.038	<0.001	<0.001	0.246
NEg intake, Mcal/day	9.49	9.90	9.65	9.54	0.272	0.682	0.880	0.264
FCR	13.92	13.24	14.07	12.69	0.477	0.130	0.162	0.505

^1^ BW, body weight; ADG, average daily gain; DMI, dry matter intake; CP, crude protein; NEg, net energy for growth; FCR, feed conversion ratio, DMI (g)/ADG (g). ^2^ LCP, low crude protein (15%); MLCP, middle-low crude protein (18%); MHCP, middle-high crude protein (19%); HCP, high crude protein (21%). ^a–c^ Means that do not have common superscripts significantly differed between the treatments (*p* < 0.05).

**Table 4:** Effects of dietary crude protein level in concentrate mix on rumen characteristics.

Items ^1^	Treatment ^2^				SEM	*p*-Value		
	LCP	MLCP	MHCP	HCP		Mean	Linear	Quadratic
pH	6.71	6.61	6.72	6.73	0.115	0.871	0.813	0.503
NH_3_-N, mg/dL	3.58 ^c^	5.57 ^bc^	6.59 ^ab^	8.33 ^a^	0.679	<0.001	<0.001	0.479
Total VFA, mM	54.3	56.5	58.0	59.1	3.50	0.734	0.263	0.963
Molar proportions, mmol/mol								
Acetate	600	603	620	603	6.5	0.080	0.270	0.323
Propionate	235 ^a^	222 ^ab^	210 ^b^	215 ^ab^	6.3	0.018	0.004	0.387
Isobutyrate	21	22	20	21	2.0	0.940	0.970	0.925
Butyrate	106 ^b^	115 ^ab^	118 ^ab^	122 ^a^	4.1	0.029	0.003	0.909
Isovalerate	24	23	19	21	2.1	0.305	0.160	0.824
Valerate	13 ^b^	15 ^ab^	13 ^b^	17 ^a^	0.6	<0.001	0.001	0.186
Acetate/propionate	2.6	2.8	3.0	2.8	0.1	0.058	0.030	0.091

^1^ VFA, volatile fatty acid. ^2^ LCP, low crude protein (15%); MLCP, middle-low crude protein (18%); MHCP, middle-high crude protein (19%); HCP, high crude protein (21%). ^a–c^ Means that do not have common superscripts significantly differed between the treatments (*p* < 0.05).

**Table 5:** Effects of dietary crude protein level in concentrate mix on blood metabolites.

Items ^1^	Treatment ^2^				SEM	*p*-Value		
	LCP	MLCP	MHCP	HCP		Mean	Linear	Quadratic
Total protein, g/dL	6.3	6.2	6.1	6.1	0.11	0.330	0.080	0.924
Urea, mg/dL	13.3 ^b^	16.3 ^ab^	19.6 ^a^	20.5 ^a^	1.21	0.001	<0.001	0.917
Glucose, mg/dL	78.8	77.8	77.0	77.5	1.81	0.892	0.507	0.770
NEFA, mEq/L	0.47 ^a^	0.37 ^b^	0.34 ^b^	0.36 ^b^	0.024	0.002	<0.001	0.078
Albumin, mg/dL	3.4	3.5	3.5	3.5	0.06	0.871	0.425	0.856
Creatinine, mg/dL	1.4	1.3	1.2	1.2	0.08	0.457	0.126	0.884
Triglyceride, mg/dL	14.8	21.0	17.3	15.8	2.18	0.205	0.688	0.054
GOT, U/L	57.9	54.4	54.4	55.8	3.41	0.843	0.562	0.505
GPT, U/L	19.3	17.8	19.4	20.7	1.09	0.318	0.366	0.113
Cholesterol, mg/dL	323.6 ^a^	277.6 ^ab^	275.2 ^ab^	235.9 ^b^	19.30	0.020	0.003	0.803
Calcium, mg/dL	9.0	8.9	8.6	8.8	0.11	0.156	0.085	0.840
Phosphorus, mg/dL	6.4	7.0	6.7	6.7	0.18	0.135	0.239	0.056

^1^ NEFA, non-esterified fatty acid; GOT, glutamic oxaloacetic transaminase; GPT, glutamic pyruvic transaminase. ^2^ LCP, low crude protein (12%); MLCP, middle-low crude protein (15.5%); MHCP, middle-high crude protein (17.25%); HCP, high crude protein (19%). ^a–b^ Means that do not have common superscripts significantly differed between the treatments (*p* < 0.05).

**Table 6:** Effects of dietary crude protein level in concentrate mix on methane emission.

Items ^1^	Treatment ^2^				SEM	*p*-Value		
	LCP	MLCP	MHCP	HCP		Mean	Linear	Quadratic
CH_4_ from eructation								
CH_4_ ppm	161.8 ^ab^	170.8 ^a^	155.0 ^ab^	126.3 ^b^	13.10	0.043	0.063	0.045
CH_4_ ppm/kg of DMI	18.3	18.0	16.1	13.5	1.41	0.030	0.014	0.144
CH_4_ ppm/kg of FNDFI	104.9 ^a^	104.9 ^a^	82.5 ^ab^	72.5 ^b^	8.94	0.010	0.005	0.158
CH_4_ ppm/kg of NDFI	49.2 ^a^	47.1 ^a^	40.3 ^ab^	33.7 ^b^	3.72	0.007	0.002	0.163
CH_4_ ppm/kg of ADG	271.1 ^a^	254.7 ^a^	240.6 ^ab^	179.3 ^b^	21.53	0.015	0.008	0.111
CH_4_ from respiration								
CH_4_ ppm	27.9	30.9	31.6	25.9	2.40	0.167	0.831	0.049
CH_4_ ppm/kg of DMI	3.2	3.3	3.3	2.7	0.25	0.213	0.324	0.124
CH_4_ ppm/kg of FNDFI	18.0	18.9	16.8	14.6	1.44	0.097	0.086	0.100
CH_4_ ppm/kg of NDFI	8.5	8.5	8.2	6.8	0.63	0.097	0.081	0.123
CH_4_ ppm/kg of ADG	46.7 ^ab^	46.1 ^ab^	48.9 ^a^	36.6 ^b^	3.48	0.035	0.118	0.067

Two steers whose forage intakes during the methane measurement period were unknown were excluded from the analysis. ^1^ CH_4_, Methane; DMI, dry matter intake; FNDFI, forage neutral detergent fiber intake; NDFI, neutral detergent fiber intake. ^2^ LCP, low crude protein (15%); MLCP, middle-low crude protein (18%); MHCP, middle-high crude protein (19%); HCP, high crude protein (21%). ^a–b^ Means that do not have common superscripts significantly differed between the treatments (*p* < 0.05).

## Data Availability

The data presented in this study are available on request from the corresponding author. The data are not publicly available due to privacy and confidentiality.
